# Error Exponents of LDPC Codes under Low-Complexity Decoding

**DOI:** 10.3390/e23020253

**Published:** 2021-02-22

**Authors:** Pavel Rybin, Kirill Andreev, Victor Zyablov

**Affiliations:** 1 Center for Computational and Data-Intensive Science and Engineering, Skolkovo Institute of Science and Technology, 121205 Moscow, Russia; k.andreev@skoltech.ru; 2 Sirius University of Science and Technology, 1 Olympic Ave, 354340 Sochi, Russia; 3 Laboratory №3—Transmission, Protection and Analysis of Information, Institute for Information Transmission Problems, Russian Academy of Sciences, 119991 Moscow, Russia; zyablov@iitp.ru

**Keywords:** low-density parity check (LDPC) codes, Gallager’s LDPC codes, binary LDPC codes, decoding algorithm, low-complexity, error exponent, capacity

## Abstract

This paper deals with the specific construction of binary low-density parity-check (LDPC) codes. We derive lower bounds on the error exponents for these codes transmitted over the memoryless binary symmetric channel (BSC) for both the well-known maximum-likelihood (ML) and proposed low-complexity decoding algorithms. We prove the existence of such LDPC codes that the probability of erroneous decoding decreases exponentially with the growth of the code length while keeping coding rates below the corresponding channel capacity. We also show that an obtained error exponent lower bound under ML decoding almost coincide with the error exponents of good linear codes.

## 1. Introduction

Low-density parity-check (LDPC) codes [1] are known for their very efficient low-complexity decoding algorithms. This paper’s central question is: Are there LDPC codes that asymptotically achieve the capacity of binary-symmetric channel (BSC) under a low-complexity decoding algorithm? The following results help us construct LDPC code with specific construction and develop a decoding algorithm to answer yes to this question. So, Zyablov and Pinsker showed in [2] that the ensemble of LDPC codes, proposed by Gallager (G-LDPC codes), includes codes that can correct a number of the errors that grow linearly with the code length *n* while the decoding complexity remains $O\left(n\log n\right)$. Later the lower bound on this fraction of errors was improved in [3,4,5]. Thus, the main idea of LDPC code construction and decoding algorithm, considered in this paper, is as follows. We need to introduce to the construction of G-LDPC code some “good” codes that reduce the number of errors from the channel in such a way that the low-complexity majority decoding can correct the rest errors. As “good” codes, we select the codes with the error exponent of good codes under ML decoding [6]. To introduce these “good” codes to the construction, we compose the parity-check matrix layer with the parity-check matrices of “good” codes. To meet the requirements on low-complexity ($O\left(n\log n\right)$) of decoding algorithm we impose restrictions on the length of “good” codes (it must be small (loglog(n)) compared to the length of whole construction). To show that the proposed construction asymptotically achieves the capacity of BSC we consider the estimation on error-exponent under the proposed low-complexity decoding algorithm.

It worth mention that papers [7,8] introduce expander codes achieving the BSC capacity under an iterative decoding algorithm with low complexity. But in this paper, we are interested in LDPC-code construction and corresponding decoding algorithm.

To show that the proposed construction of LDPC code is good, we also estimate the error exponent under ML decoding and compare it with the error exponent under the proposed low-complexity decoding algorithm. Previously the authors of [9,10] have derived the upper and lower bounds on the G-LDPC codes error exponent under the ML decoding assumption. Moreover, one can conclude from [10] that the lower bound on the error exponent under ML decoding of G-LDPC codes almost coincides with the lower bound obtained for good linear codes (from [6]) under ML.

Some parts of this paper were previously presented (with almost all of the proofs omitted) in the conference paper [11]. The low-complexity decoding algorithm that we use for our analysis was proposed in [12,13]. Unlike in previous papers, Corollary 1 is significantly enhanced and proved in detail in this paper. Moreover, the results for the error-exponent bound under ML decoding and corresponding proofs are added. We compare the obtained lower bounds on the error exponents under the low-complexity decoding and the ML decoding. We evaluate the error exponents numerically for different code parameters.

## 2. LDPC Code Construction

Let us briefly consider the LDPC code construction from [11,12]. First, let us consider the G-LDPC code parity-check matrix $H_{2}$ of size $\ell \times b_{0}n_{0}$ from [1]:

$$H_{2}=\left(\begin{array}{c} \pi_{1}\left(H_{b_{0}}\right)\\ \pi_{2}\left(H_{b_{0}}\right)\\ \vdots \\ \pi_{\ell }\left(H_{b_{0}}\right) \end{array}\right).$$

Here we denote $\pi_{l}\left(H_{b_{0}}\right)$, l=1,⋯,ℓ, as a random column permutation of $H_{b_{0}}$, which is given by

$$H_{b_{0}}=\left(\underset{b_{0}}{\underset{︸}{\begin{array}{cccc} H_{0} & 0 & \ldots & 0\\ 0 & H_{0} & \ldots & 0\\ \vdots & \vdots & \ddots & \vdots \\ 0 & 0 & \ldots & H_{0} \end{array}}}\right),$$

where $H_{0}$ is the parity-check matrix of the constituent single parity check (SPC) code of length $n_{0}$.

The elements of the Gallager’s LDPC codes ensemble $E_{G}\left(\ell ,n_{0},b_{0}\right)$ are obtained by independently selecting the equiprobable permutations $\pi_{l}$, *l* = 1, 2, …, *ℓ*.

One can write a lower bound on the G-LDPC code rate $E_{G}\left(\ell ,n_{0},b_{0}\right)$ as

(1) $$R_{2}\geq 1-\ell \left(1-R_{0}\right),$$

where $R_{0}=\frac{n_{0}-1}{n_{0}}$ is a SPC code rate.

The equality achieved if and only if the matrix $H_{2}$ has full rank.

Consider a G-LDPC parity check matrix with an additional layer consisting of linear codes (LG-LDPC code). Let us denote this matrix as H:

$$H=\left(\begin{array}{c} \pi_{1}\left(H_{b_{0}}\right)\\ \pi_{2}\left(H_{b_{0}}\right)\\ \vdots \\ \pi_{\ell }\left(H_{b_{0}}\right)\\ \pi_{\ell +1}\left(H_{b_{1}}\right) \end{array}\right),$$

where $H_{b_{1}}$ is given by

$$H_{b_{1}}=\left(\underset{b_{1}}{\underset{︸}{\begin{array}{cccc} H_{1} & 0 & \ldots & 0\\ 0 & H_{1} & \ldots & 0\\ \vdots & \vdots & \ddots & \vdots \\ 0 & 0 & \ldots & H_{1} \end{array}}}\right),$$

where $b_{1}$ is such that $b_{1}n_{1}=b_{0}n_{0}$. As soon as the first *ℓ* layers of matrix H is the G-LDPC parity-check matrix, we can write H as

$$H=\left(\begin{array}{c} H_{2}\\ \pi_{\ell +1}\left(H_{b_{1}}\right) \end{array}\right).$$

For a given SPC code with the code length $n_{0}$ and the parity-check matrix $H_{0}$ and for a given linear code with the code length $n_{1}$ and the parity-check matrix $H_{1}$, the elements of the LG-LDPC codes ensemble $E_{LG}\left(\ell ,n_{0},b_{0},R_{1},n_{1},b_{1}\right)$ are obtained by independently selecting the equiprobable permutations $\pi_{l}$, *l* = 1, 2, …, ℓ+1.

The length of the constructed LG-LDPC code is $n=b_{0}n_{0}=b_{1}n_{1}$, and the code rate *R* is lower bounded by

$$R\geq R_{1}-\ell \left(1-R_{0}\right),$$

According to (1),

$$R\geq R_{1}+R_{2}-1.$$

## 3. Decoding Algorithms

In this paper, we consider two decoding algorithms of the proposed construction. The first one is the well-known maximum likelihood decoding algorithm $A_{ML}$. Under the second decoding algorithm $A_{C}$, the LG-LDPC code is decoded as a concatenated code. In other words, in the first step, we decode the received sequence using linear codes with the parity-check matrix $H_{1}$ from the ℓ+1 layer of H. Then, in the second step, we decode the sequence obtained in the previous step using the G-LDPC code with the parity-check matrix $H_{2}$. Thus, this algorithm $A_{C}$ consists of the following two steps:

1. The received sequence is separately decoded with the well-known maximum likelihood algorithm by $b_{1}$ linear codes with the parity-check matrix $H_{1}$ from the ℓ+1 layer of H.
2. The tentative sequence is decoded with the well-known bit-flipping (majority) decoding algorithm $A_{M}$ by the G-LDPC code with the parity-check matrix $H_{2}$.

An important note here is that $A_{C}$ is a two-step decoding algorithm, and each step is performed only once. It first decodes the received sequence once by the ML decoding algorithm using linear codes $H_{1}$. Then it applies the iterative bit-flipping (majority) algorithm $A_{M}$ using the G-LDPC code to the tentative sequence.

It is also worth noting that the complexity of the proposed decoding algorithm $A_{C}$ is $O\left(n\log n\right)$ with some restrictions on the length of the linear codes with the parity-check matrix $H_{1}$ (see Lemma 3). At the same time the complexity of ML decoding is exponential.

## 4. Main Results

Consider a BSC with a bit error probability *p*. Let a decoding error probability *P* be the probability of the union of decoding denial and the erroneous decoding events. In this paper, we consider the decoding error probability *P* in the following form

$$P\leq \exp\left\{-nE\left(\cdot \right)\right\},$$

with the $E\left(\cdot \right)$ being the required error exponent.

Let us define two error exponents: $E_{C}\left(\cdot \right)$ and $E_{ML}\left(\cdot \right)$ corresponding to the $A_{C}$ decoding algorithm (having $O\left(n\log n\right)$ complexity) and the $A_{ML}$ decoding algorithm (having an exponential complexity) respectively. Let us consider first the error exponent $E_{C}\left(\cdot \right)$.

**Theorem** **1.** *Let there exist in the ensemble $E_{G}\left(\ell ,n_{0},b_{0}\right)$ of the G-LDPC codes a code with the code rate $R_{2}$ that can correct any error pattern of weight up to $\left\lfloor \omega_{t}n\right\rfloor$ while decoding with the bit-flipping (majority) algorithm $A_{M}$.*

*Let there exist a linear code with code length $n_{1}$, code rate $R_{1}$ and an error exponent under maximum likelihood decoding lower bounded by $E_{0}\left(R_{1},p\right)$.*

*Then, in the ensemble $E_{LG}\left(\ell ,n_{0},b_{0},R_{1},n_{1},b_{1}\right)$ of the LG-LDPC codes, there exists a code with code length n,*

$$n=n_{0}b_{0}=n_{1}b_{1},$$

*code rate R,*

$$R\geq R_{1}+R_{2}-1,$$

*and an error exponent over the memoryless BSC with BER p under the decoding algorithm $A_{C}$ with complexity $O\left(n\log n\right)$ lower bounded by $E_{C}\left(\cdot \right)$:*

(2) $$E_{C}\left(R_{1},n_{1},\omega_{t},p\right)=\min_{\omega_{t}\leq \beta \leq \beta_{0}}\left\{\beta E_{0}\left(R_{1},p\right)+E_{2}\left(\beta ,\omega_{t},p\right)-\frac{1}{n_{1}}H\left(\beta \right)\right\},$$

*where $\beta_{0}=\min\left(\frac{\omega_{t}}{2p},1\right)$, $H\left(\beta \right)=-\beta \ln\beta -\left(1-\beta \right)\ln\left(1-\beta \right)$ – an entropy function – and $E_{2}\left(\beta ,\omega_{t},p\right)$ is given by*

$$E_{2}\left(\beta ,\omega_{t},p\right)=\frac{1}{2}\left(\omega_{t}\ln\frac{\omega_{t}}{p}+\left(2\beta -\omega_{t}\right)\ln\frac{2\beta -\omega_{t}}{1-p}\right)-\beta \ln\left(2\beta \right),$$

*where $n_{1}$ satisfies the following condition:*

(3) $$\frac{-\ln\beta_{0}}{E_{0}\left(R_{1},p\right)}\leq n_{1}\leq \frac{1}{R_{1}}\log_{2}\log_{2}\left(n\right).$$

**Corollary** **1.** *$E_{C}\left(\cdot \right)>0$, if R→C, where C is the capacity of a memoryless BSC with error probability p, such that $R_{1}\to C$ and $R_{2}<1$.*

Thus, according to Corollary 1, we can state that there exists an LG-LDPC code such that the error probability of the low-complexity decoding algorithm $A_{C}$ exponentially decreases with the code length for all code rates below the channel capacity *C*.

**Remark** **1.** *We have obtained the lower bound on $E_{C}\left(R_{1},n_{1},\omega_{t},p\right)$ assuming n→∞, where $n_{0}=const$, $n_{1}=const$, $b_{0}\to \infty$, and $b_{1}\to \infty$. As a result, the complexity of $A_{C}$ algorithm equals to $O\left(n\log n\right)$, and we have the right inequality of condition (3) for $n_{1}$.*

Theorem 1 was obtained in [12]. The main idea of the proof is based on the following results. Our previous results [3,4] show that in the ensemble $E_{G}\left(\ell ,n_{0},b_{0}\right)$ of G-LDPC codes, there exists a code that can correct any error pattern of weight up to $\left\lfloor \omega_{t}n\right\rfloor$ under the algorithm $A_{M}$ with complexity $O\left(n\log n\right)$. In [6], it was shown that there exists a linear code for which the error exponent under ML decoding is lower bounded by $E_{0}\left(R,p\right)$, where $E_{0}\left(R,p\right)>0$ for ∀R<C.

Let us now consider the lower bound on the error exponent $E_{ML}\left(\cdot \right)$.

**Theorem** **2.** *In the ensemble $E_{LG}\left(\ell ,n_{0},b_{0},R_{1},n_{1},b_{1}\right)$, there exists an LG-LDPC code such that the error exponent of this code over the memoryless BSC with BER p under the decoding algorithm $A_{ML}$ is lower bounded by*

$$E_{ML}\left(p\right)=\max_{\omega_{0}\leq \omega_{c}\leq 1}\left\{\min\left(E_{\delta }\left(\omega_{c},p\right),E_{\omega_{c}}\left(\omega_{c},p\right)\right)\right\},$$

*where $\omega_{0}=\max\left(\delta ,p\right)$, δ is the code distance of this code, and $E_{\delta }\left(\omega_{c},p\right)$ is given by*

$$E_{\delta }\left(\omega_{c},p\right)=\max_{\delta \leq \omega \leq \omega_{c}}\left\{\nu \left(\omega \right)+\omega \left(\ln2+\ln\sqrt{p\left(1-p\right)}\right)\right\},$$

*where $\nu \left(\omega \right)$ is an asymptotic spectrum of the LG-LDPC code:*

$$\nu \left(\omega \right)=\lim_{n\to \infty }\frac{\ln\bar{N}\left(\omega n\right)}{n},$$

*where $\bar{N}\left(\omega n\right)$ is the average number of codewords and $E_{\omega_{c}}\left(\omega_{c},p\right)$ is given by*

$$E_{\omega_{c}}\left(\omega_{c},p\right)=\left(1-\omega_{c}\right)\ln\frac{1-\omega_{c}}{1-p}+\omega_{c}\ln\frac{\omega_{c}}{p}.$$

We have obtained Theorem 2 using the methods developed in [14] to estimate the error exponent under the ML decoding of codes with the given spectrum. We have taken ideas of [1] for G-LDPC codes to construct the upper bound on the code spectrum and the lower bound on the code distance of the proposed LDPC construction (see Lemmas 1 and 2).

**Lemma** **1.** *The value of $\nu \left(\omega \right)$ for the codes from the ensemble $E_{LG}\left(\ell ,n_{0},b_{0},R_{1},n_{1},b_{1}\right)$ of LG-LDPC codes is upper bounded by*

$$\nu \left(\omega \right)\leq \nu_{0}\left(\omega \right)=-\left(\ell -1\right)H\left(\omega \right)+\min_{s>0}\left\{\frac{\ell -1}{n_{0}}\ln g_{0}\left(s,n_{0}\right)+\frac{1}{n_{1}}\ln g_{1}\left(s,R_{1},n_{1}\right)-\omega \ell \ln s\right\},$$

*where $g_{0}\left(s,n_{0}\right)$ is a spectrum function of the constituent SPC code with length $n_{0}$,*

$$g_{0}\left(s,n_{0}\right)=\sum_{i=0}^{n_{0}}\frac{{\left(1+s\right)}^{n_{0}}+{\left(1-s\right)}^{n_{0}}}{2},$$

*and $g_{1}\left(s,R_{1},n_{1}\right)$ is a spectrum function of the constituent linear code with a good spectrum, code rate $R_{1}$ and length $n_{1}$ obtained in [14]:*

$$g_{1}\left(s,R_{1},n_{1}\right)\leq 1+n_{1}2^{-\left(1-R_{1}\right)n_{1}}\sum_{i=\left\lceil \delta_{VG}n_{1}\right\rceil }^{n_{1}}\left(\begin{array}{c} n_{1}\\ i \end{array}\right)s^{i},$$

*where $\delta_{VG}$ is given by the Varshamov-Gilbert bound.*

**Lemma** **2.** *Let the positive root $\delta_{0}$ of the following equation exist:*

$$\nu_{0}\left(\delta_{0}\right)=0.$$

*Then, a code with minimum code distance $\delta \geq \delta_{0}$ exists in ensemble $E_{LG}\left(\ell ,n_{0},b_{0},R_{1},n_{1},b_{1}\right)$.*

## 5. Numerical Results

One can see from Theorems 1 and 2 that the obtained lower-bounds $E_{C}\left(\cdot \right)$ and $E_{ML}\left(\cdot \right)$ depend on the set of parameters: error probability *p* of BSC, code rate $R_{1}$ and length $n_{1}$ of linear code from added layer, code rate $R_{2}$ and constituent code length $n_{0}$ of G-LDPC code (the value of $\omega_{t}$, used in $E_{C}\left(\cdot \right)$ bound, depends on these parameters), wherein the code rate *R* of whole construction depends on $R_{1}$ and $R_{2}$.

**Figure 1:**
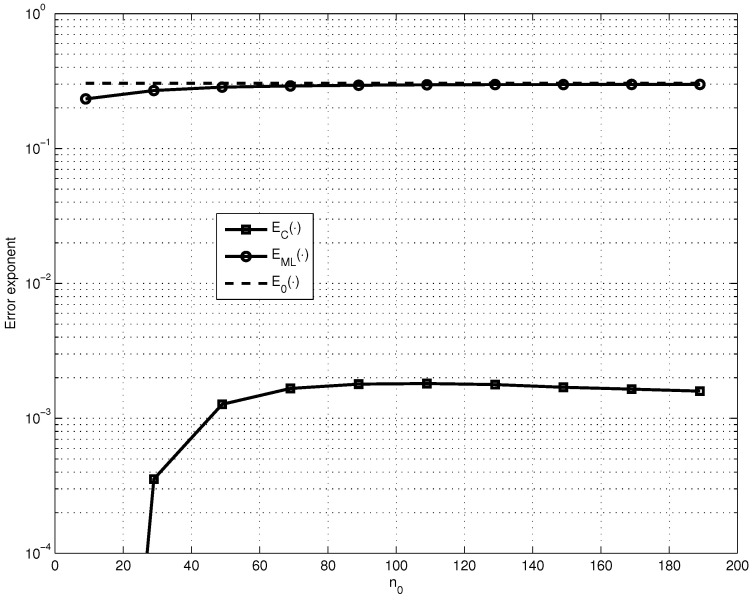
Comparison of the dependence on $n_{0}$ of $E_{C}\left(\cdot \right)$, $E_{ML}\left(\cdot \right)$ and $E_{0}\left(\cdot \right)$ for fixed $R_{1}\approx 0.85$, $n_{1}=2000$, R=0.5 and $p=10^{-3}$.

**Figure 2:**
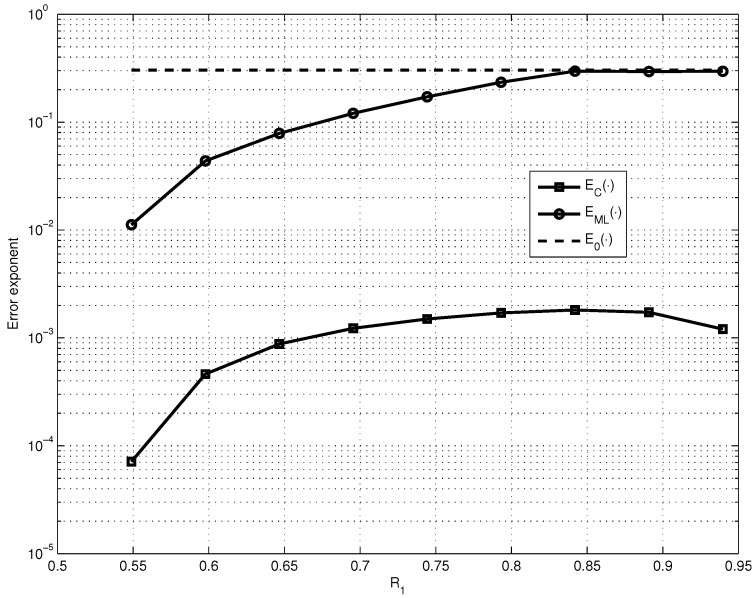
Comparison of the dependences on $R_{1}$ of $E_{C}\left(\cdot \right)$, $E_{ML}\left(\cdot \right)$ and $E_{0}\left(\cdot \right)$ for fixed $n_{1}=2000$, R=0.5 and $p=10^{-3}$.

Thus, to simplify the analysis let us first fix the parameters $R_{1}=0.85$, $n_{1}=2000$, R=0.5 and $p=10^{-3}$ and find how $E_{C}\left(\cdot \right)$ and $E_{ML}\left(\cdot \right)$ depend on the SPC code length $n_{0}$ (see Figure 1). Then, let us consider the maximized $E_{ML}\left(\cdot \right)$ and $E_{C}\left(\cdot \right)$ over the values of $n_{0}$ (see Figure 2).

We can explain the different behaviors of $E_{ML}\left(\cdot \right)$ and $E_{C}\left(\cdot \right)$ shown in Figure 1 and Figure 2 by the following: the value of $E_{ML}\left(\cdot \right)$ significantly depends on the value of the code distance δ of the LG-LDPC code and the value of $E_{C}\left(\cdot \right)$ depends on the value of the error fraction $\omega_{t}$, which is guaranteed to be corrected by the G-LDPC code. And it is known that for the fixed code rate *R* code distance of LDPC code increases with the growth of constituent code length $n_{0}$ and guaranteed corrected error fraction $\omega_{t}$ has the maximum for the certain parameters $n_{0}$ and *ℓ*.

**Figure 3:**
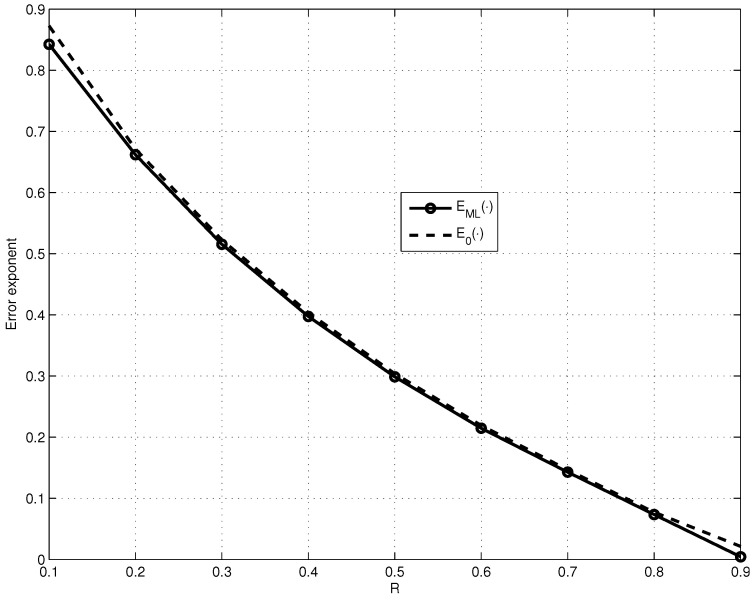
Comparison of the dependencies on *R* for fixed $p=10^{-3}$ of $E_{ML}\left(\cdot \right)$, maximized over the values of $n_{0}$ and $R_{1}$ for fixed $n_{1}=2000$, and of $E_{0}\left(\cdot \right)$.

In Figure 3, we compare the dependencies on *R* for fixed $p=10^{-3}$ of the obtained lower bound $E_{ML}\left(\cdot \right)$, maximized over the values of $n_{0}$ and $R_{1}$, and of the lower bound $E_{0}\left(\cdot \right)$.

**Figure 4:**
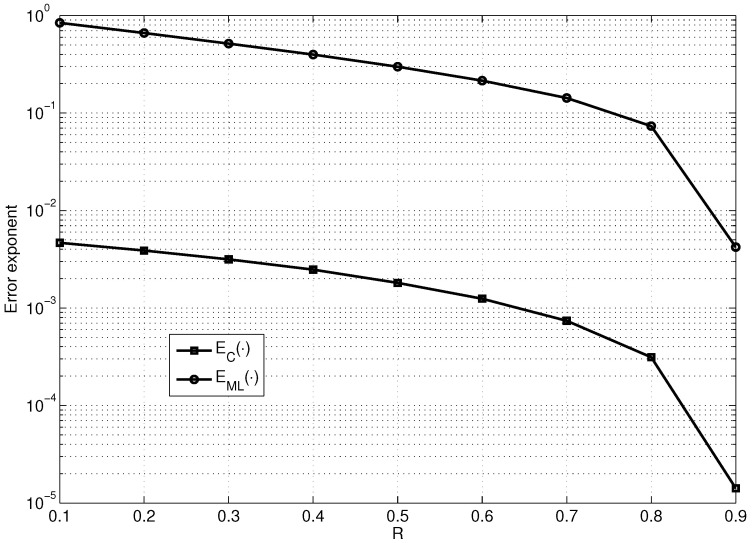
Comparison of the dependencies on *R* of $E_{C}\left(\cdot \right)$ and $E_{ML}\left(\cdot \right)$, maximized over the values of $n_{0}$ and $R_{1}$ for fixed $n_{1}=2000$ and $p=10^{-3}$.

Figure 4 shows the dependencies on *R* of the maximum values of $E_{ML}\left(\cdot \right)$ and $E_{C}\left(\cdot \right)$ for fixed $p=10^{-3}$ (the maximization was performed over the values of $n_{0}$ and $R_{1}$).

As observed from Figure 4, $E_{C}\left(\cdot \right)$ is approximately two orders of magnitude smaller than $E_{ML}\left(\cdot \right)$, which almost reaches the lower bound on the error exponent $E_{0}\left(\cdot \right)$ of the good linear code (see Figure 3). However, it is important to note that $E_{ML}\left(\cdot \right)$ encounters only exponential decoding complexity and $E_{C}\left(\cdot \right)$ encounters the decoding complexity of $O\left(n\log n\right)$.

## 6. Conclusions

The main result of this paper is that we prove (see Corollary 1) the existence of such LDPC code with specific construction that the probability of erroneous decoding with low-complexity algorithm ($O\left(n\log n\right)$) decreases exponentially with the growth of the code length for all code rates below BSC capacity. We also obtain the lower bound on error exponent under ML decoding for proposed construction (see Theorem 2) and show with numeric results that obtained lower-bound almost coincide with the error exponents of good linear codes for the certain parameters.

As a future work to improve the lower-bound for the low-complexity decoder, we plan to consider error-reducing codes instead of good linear codes and generalize our results for channels with reliability (e.g., channels with additive white Gaussian noise (AWGN) and “soft” reception).

## 7. Proofs of the Main Results

### 7.1. Error Exponent for Decoding Algorithm $A_{C}$

Theorem 1 was proved in [12]. Here, we provide the proof for convenience of the reader in more detail, especially for the essential Corollary 1.

Let us first consider the complexity of the decoding algorithm $A_{C}$ of an LG-LDPC code.

**Lemma** **3.** *The complexity of the decoding algorithm $A_{C}$ of an LG-LDPC code with length n is of order O(nlogn) if the length of the linear code satisfies the inequality $n_{1}\leq \frac{1}{R_{1}}\log_{2}\log_{2}\left(n\right)$.*

**Proof.**  Since the length of the linear code is equal to $n_{1}$ and the code rate is $R_{1}$, the complexity of the maximum likelihood decoding algorithm for the single code is of order $O(2^{R_{1}n_{1}})$. The total number of codes is $b_{1}$, which is proportional to *n*, and then, the complexity of decoding all of the codes is of order $O(n2^{R_{1}n_{1}})$.

In [4], it was shown that the complexity of the bit-flipping decoding algorithm of LDPC codes is O(nlogn).

Therefore, the complexity of decoding algorithm $A_{C}$ is of order $O(n\log_{2}n)$ if the following condition is satisfied:

$$n2^{R_{1}n_{1}}\leq n\log_{2}\left(n\right).$$

Here we find the condition on $n_{1}$:

(4) $$n_{1}\leq \frac{1}{R_{1}}\log_{2}\log_{2}\left(n\right).$$

 □

Let us now consider the proof of Theorem 1.

**Proof.**  Assume that in the first step of the decoding algorithm $A_{C}$ of LG-LDPC code, the decoding error occurred exactly in *i* linear codes. Since each code contains no more than $n_{1}$ errors, the total number of errors *W* after the first step of decoding is no greater than $in_{1}$. Let $i=\beta b_{1}$, where β is the fraction of linear codes in which the decoding failure occurred; then,

$$W\leq \beta b_{1}n_{1}=\beta n.$$

According to [4], LDPC code is capable of correcting any error pattern with weight less than *W*, that is,

$$W<W_{0}=\left\lfloor \omega_{t}n\right\rfloor ,$$

where $\omega_{t}$ is the fraction of errors guaranteed corrected by the G-LDPC code [4] (Theorem 1). Consequently, for the case where $\beta <\omega_{t}$, the decoding error probability *P* for LG-LDPC code under decoding algorithm $A_{C}$ is equal to 0:

$$P=0,\;\beta <\omega_{t}.$$

At $\beta >\omega_{t}$, the error decoding probability is defined as

(5) $$P=\sum_{i=\left\lfloor \omega_{t}b_{1}\right\rfloor }^{b_{1}}\left(\begin{array}{c} b_{1}\\ i \end{array}\right)P_{2}\left(W\geq W_{0}|i\right)P_{1}^{i}{\left(1-P_{1}\right)}^{b_{1}-i},$$

where $P_{1}$ is the error decoding probability of linear code,

$$P_{1}\leq \exp\left\{-n_{1}E_{0}\left(R_{1},p\right)\right\},$$

and $P_{2}\left(W\geq W_{0}|i\right)$ is the probability that the number of errors after the first step of the decoding algorithm $A_{C}$ is not less than $W_{0}$ under the condition that the decoding error occurred exactly in *i* linear codes.

Since the number of errors no more than doubles in a block in the case of error decoding with the maximum likelihood decoding algorithm, it must be greater than $\frac{W_{0}}{2}$ errors before the first step in *i* erroneous blocks to have more than $W_{0}$ errors after the first step of decoding algorithm $A_{C}$. Then, we can write $P_{2}\left(W\geq W_{0}|i\right)$ as

$$P_{2}\left(W\geq W_{0}|i\right)=\sum_{j=\left\lfloor \frac{\omega_{t}n}{2}\right\rfloor }^{in_{1}}\left(\begin{array}{c} in_{1}\\ j \end{array}\right)p^{j}{\left(1-p\right)}^{in_{1}-j}.$$

Using the Chernoff bound, we obtain

$$P_{2}\left(W\geq W_{0}|i\right)\leq \exp\left\{-nE_{2}\left(\beta ,\omega_{t},p\right)\right\},$$

where

(6) $$E_{2}\left(\beta ,\omega_{t},p\right)=\left\{\begin{array}{c} \frac{1}{2}\left(\omega_{t}\ln\frac{\omega_{t}}{p}+\left(2\beta -\omega_{t}\right)\ln\frac{2\beta -\omega_{t}}{1-p}\right)-\beta \ln2\beta ,\;\beta <\beta_{0},\\ 0,\;\beta \geq \beta_{0}. \end{array}\right.$$

Here $\beta =\frac{i}{b_{1}}>\omega_{t}$, and

$$\beta_{0}=\min\left(\frac{\omega_{t}}{2p},1\right)$$

because β>1 has no sense.

In accordance with (6), the probability $P_{2}\left(W\geq W_{0}|i\right)$ can be replaced with the trivial estimation $P_{2}\left(W\geq W_{0}|i\right)\leq 1$ for $i\geq \lceil \beta_{0}b_{1}\rceil$, and then, sum (5) is upper bounded as follows:

$$P⩽\sum_{i=\left\lfloor \omega_{t}b_{1}\right\rfloor }^{\left\lfloor \beta_{0}b_{1}\right\rfloor }\left(\begin{array}{c} b_{1}\\ i \end{array}\right)P_{2}\left(W\geq W_{0}|i\right)P_{1}^{i}{\left(1-P_{1}\right)}^{b_{1}-i}+\sum_{i=\left\lceil \beta_{0}b_{1}\right\rceil }^{b_{1}}\left(\begin{array}{c} b_{1}\\ i \end{array}\right)P_{1}^{i}{\left(1-P_{1}\right)}^{b_{1}-i}.$$

Let $P_{\mathrm{II}}$ denote the first sum in the right part of this inequality and $P_{I}$ denote the second sum. Let us consider each sum separately.

The sum $P_{I}$ can be easily estimated as a tail of the binomial distribution with probability $P_{1}$ using the Chernoff bound:

$$P_{I}\leq \exp\left\{-nE_{I}\left(R_{1},n_{1},\omega_{t},p\right)\right\},$$

where

$$E_{I}\left(R_{1},n_{1},p\right)=\beta_{0}E_{0}\left(R_{1},p\right)-\frac{1}{n_{1}}H\left(\beta_{0}\right),$$

and $P_{1}$ satisfies the condition

$$P_{1}\left(n_{1},R_{1},p\right)\leq \beta_{0}.$$

Thus,

(7) $$n_{1}\geq \frac{-\ln\beta_{0}}{E_{0}\left(R_{1},p\right)}.$$

Let us now consider the sum $P_{\mathrm{II}}$:

$$P_{II}\leq \left\lceil \left(\beta_{0}-\omega_{t}\right)b_{1}\right\rceil \times \max_{\omega_{t}\leq \beta \leq \beta_{0}}\left\{\left(\begin{array}{c} b_{1}\\ \beta b_{1} \end{array}\right)P_{2}\left(W\geq W_{0}|\beta b_{1}\right)P_{1}^{\beta b_{1}}{\left(1-P_{1}\right)}^{\left(1-\beta \right)b_{1}}\right\}.$$

Hence, at n→∞ ($b_{1}\to \infty$ and $b_{0}\to \infty$), we obtain

(8) $$E_{\mathrm{II}}\left(R_{1},n_{1},\omega_{t},p\right)=\min_{\omega_{t}\leq \beta \leq \beta_{0}}\left\{E_{2}\left(\beta ,\omega_{t},p\right)+\beta E_{0}\left(R_{1},p\right)-\frac{1}{n_{1}}H\left(\beta \right)\right\}.$$

Let us note that if a minimum is achieved at $\beta_{0}$ in the right part of equality (8), then according to (6), we obtain $E_{\mathrm{II}}=E_{I}$. Consequently, $E_{\mathrm{II}}\leq E_{I}$.

It is easy to see that at n→∞, the following inequality is satisfied:

$$P\leq \exp\{-nE\left(R_{1},n_{1},\omega_{t},p\right)\},$$

where $E(R_{1},n_{1},\omega_{t},p)$ = $\min\{E_{\mathrm{II}},E_{I}\}$ = $E_{\mathrm{II}}$.

According to the proved lemma, the complexity of the decoding algorithm $A_{C}$ is of order O(nlogn) if the condition (4) is satisfied, but for the obtained estimation, the condition (7) must also be satisfied. Thus,

$$\frac{-\ln\beta_{0}}{E_{0}\left(R_{1},p\right)}\leq n_{1}\leq \frac{1}{R_{1}}\log_{2}\log_{2}n.$$

This completes the proof.  □

Before proving Corollary 1, we need to consider the lower-bound behavior of the error fraction $\omega_{t}$ guaranteed corrected by G-LDPC code. In [4], the new estimation of the error fraction $\omega_{t}$ guaranteed corrected by generalized LDPC code with a given constituent code was obtained. Let us formulate this result for G-LDPC code:

**Theorem** **3.** *Let the root $\omega_{0}$ exist for the following equation:*

(9) $$h\left(\omega_{0}\right)-\ell F_{e}\left(\omega_{0},n_{0}\right)=0,$$

*where $F_{e}\left(\omega_{0},n_{0}\right)$ is given by*

$$F_{e}\left(\omega_{0},n_{0}\right)=h\left(\omega_{t}\right)+\max_{s>0,0<v<1}\left\{\omega_{0}\log_{2}sv-\frac{1}{n_{0}}\log_{2}\left(g_{e}\left(s,v,n_{0}\right)+g_{0}\left(s,n_{0}\right)\right)\right\},$$

*where $g_{0}\left(s,n_{0}\right)$ and $g_{e}\left(s,v,n_{0}\right)$ have the following forms:*

$$g_{0}\left(s,n_{0}\right)=\frac{{\left(1+s\right)}^{n_{0}}+{\left(1-s\right)}^{n_{0}}}{2},g_{e}\left(s,v,n_{0}\right)=g_{d}\left(sv^{2},n_{0}\right),$$

*where*

$$g_{d}\left(s,n_{0}\right)={\left(1+s\right)}^{n_{0}}-g_{0}\left(s,n_{0}\right).$$

*Let for the found value $\omega_{0}$, the root $\alpha_{0}$ exist for the following equation:*

(10) $$h\left(\omega_{0}\right)-\ell F_{s}\left(\alpha ,\omega_{0},n_{0},\ell \right)=0,$$

*where $F_{s}\left(\alpha ,\omega_{0},n_{0},\ell \right)$ is given by*

$$F_{s}\left(\alpha ,\omega_{0},n_{0},\ell \right)=h\left(\omega_{0}\right)+\max_{s>0,0<v<1}\left\{\omega_{0}\left(\log_{2}s+\frac{\ell -\frac{1-\alpha }{\alpha }}{\ell }\log_{2}v\right)-\frac{1}{n_{0}}\log_{2}\left(g_{d}\left(s,n_{0}\right)v+g_{0}\left(s,n_{0}\right)\right)\right\}.$$

*Then, there exists a code (with $p_{n}:\lim_{n\to \infty }p_{n}=1$) in the ensemble $E_{G}\left(\ell ,n_{0},b_{0}\right)$ of G-LDPC codes that can correct any error pattern with weight less than $\left\lfloor \omega_{t}n\right\rfloor$, where $\omega_{t}=\alpha_{0}\omega_{0}$, with decoding complexity $O\left(n\log n\right)$.*

For Theorem 3, we obtain the following:

**Corollary** **2.** *For the given code rate R<1, there exists a G-LDPC code in the ensemble $E_{G}\left(\ell ,n_{0},b_{0}\right)$ with ℓ>2 such that equation (9) has a positive root $\omega_{t}>0$.*

The proof of Theorem 3 was given in a more generalized form in [4]. Here, we consider only the proof of Corollary 2. For this purpose, let us formulate some useful facts proved in [4].

First, let us formulate the condition of the existence of a symbol that upon inversion, reduces the number of unsatisfied checks:

**Lemma** **4.** *At least one such symbol exists that will be inverted during one iteration of decoding algorithm $A_{M}$ for G-LDPC code if the following condition is satisfied:*

(11) $$E_{\sum }^{\left(W\right)}=2\sum_{j=1}^{W}e_{A_{1\to 0}}^{\left(i_{j}\right)}+\sum_{j=1}^{W}e_{A_{1\to 1}}^{\left(i_{j}\right)}>W\ell ,$$

*where W is the number of errors in the received sequence, $i_{1},i_{2},\ldots ,i_{W}$ are indices of erroneous symbols, $e_{A_{1\to 0}}^{\left(i\right)}$ is the number of edges emanating from the ith variable-node to the set of check-nodes for which the checks become satisfied after the inversion of this symbol, and $e_{A_{1\to 1}}^{\left(i\right)}$ is the number of the edges emanating from the ith variable-node to the set of check-nodes for which the checks remain unsatisfied after the inversion of this symbol.*

Now, let us consider the estimation of the probability that the above condition is not satisfied:

**Lemma** **5.** *The probability $P_{W}\left(E_{\sum }^{\left(W\right)}⩽W\ell \right)$ for the fixed pattern of errors of weight W that condition (11) is not satisfied, e.g., $E_{\sum }^{\left(W\right)}⩽W\ell$, is upper bounded as follows:*

$$P_{W}\left(E_{\sum }^{\left(W\right)}⩽W\ell \right)⩽2^{-n\ell F_{e}\left(\omega ,n_{0}\right)+o\left(n\right)},\;\omega =\frac{W}{n}.$$

Now, let us consider the proof of Corollary 2.

**Proof.**  Let us select an arbitrary small value $\varepsilon^{\prime }$ and write the following condition:

$$\lim_{n\to \infty }\sum_{W=1}^{\left\lfloor \varepsilon^{\prime }n\right\rfloor }2^{-n\left(\ell F_{e}\left(\frac{W}{n},n_{0}\right)-h\left(\frac{W}{n}\right)\right)}<1.$$

In the left part of the inequality is the upper bound on the probability of the code that condition (11) is not satisfied for some sequences.

Let us introduce the following function $G\left(\omega \right)$:

$$G\left(\omega \right)=\ell F_{e}\left(\omega ,n_{0}\right)-h\left(\omega \right)=\left(\ell -1\right)h\left(\omega \right)+\ell \max_{s>0,\;0<v<1}\left\{\omega \log_{2}sv-\frac{1}{n_{0}}\log_{2}\left(g_{e}\left(s,v,n_{0}\right)+g_{0}\left(s,n_{0}\right)\right)\right\}.$$

Since the variables *s* and *v* are dummies, they can be equal to an arbitrary value if the conditions s>0 and 0<v<1 are satisfied. Then, let us set $s=v=\sqrt[{4}]{\omega }$ (this choice is justified by the fact that due to the structure of the parity-check matrix, the conditions ℓ>2 should be satisfied):

$$G^{*}\left(\omega \right)=\left(\ell -1\right)h\left(\omega \right)+\ell \left(\frac{\omega }{2}\log_{2}\omega -\frac{1}{n_{0}}\log_{2}\left(g_{e}\left(\sqrt[{4}]{\omega },\sqrt[{4}]{\omega },n_{0}\right)+g_{0}\left(\sqrt[{4}]{\omega },n_{0}\right)\right)\right).$$

Let us transform $G^{*}\left(\omega \right)$ as follows:

$$G^{*}\left(\omega \right)=-\left(\frac{\ell }{2}-1\right)\omega \log_{2}\omega -\left(\ell -1\right)\left(1-\omega \right)\log_{2}\left(1-\omega \right)-\frac{\ell }{n_{0}}\log_{2}\left(g_{e}\left(\sqrt[{4}]{\omega },\sqrt[{4}]{\omega },n_{0}\right)+g_{0}\left(\sqrt[{4}]{\omega },n_{0}\right)\right).$$

It is easy to show that $g_{e}\left(s,v,n_{0}\right)+g_{0}\left(s,n_{0}\right)⩽{\left(1+s\right)}^{n_{0}}$ for 0<s<1 and 0<v<1. Then, we obtain

$$G^{*}\left(\omega \right)=-\left(\frac{\ell }{2}-1\right)\omega \log_{2}\omega +O\left(\omega \right).$$

It is easily noted that $G\left(\omega \right)⩾G^{*}\left(\omega \right)$ implies

$$\lim_{n\to \infty }\sum_{W=1}^{\left\lfloor \varepsilon^{\prime }n\right\rfloor }2^{-nG\left(\frac{W}{n}\right)}⩽\lim_{n\to \infty }\sum_{W=1}^{\left\lfloor \varepsilon^{\prime }n\right\rfloor }2^{-nG^{*}\left(\frac{W}{n}\right)}.$$

Since LDPC code construction requires ℓ>2, $\left(\frac{\ell }{2}-1\right)>0$, and consequently,

$$G\left(\omega \right)⩾-c_{1}\omega \log_{2}\omega +c_{2}\omega +o\left(\omega \right),\;c_{1}>0.$$

$$\lim_{n\to \infty }\sum_{W=1}^{\left\lfloor \varepsilon^{\prime }n\right\rfloor }2^{-nG\left(\frac{W}{n}\right)}⩽\lim_{n\to \infty }\sum_{W=1}^{\left\lfloor \varepsilon^{\prime }n\right\rfloor }2^{n\cdot c_{1}\cdot \frac{W}{n}\cdot \log_{2}\frac{W}{n}\;-\;n\cdot c_{2}\cdot \frac{W}{n}}=\lim_{n\to \infty }\sum_{W=1}^{\left\lfloor \varepsilon^{\prime }n\right\rfloor }{\left(\frac{W}{n}\right)}^{c_{1}W}2^{-c_{2}W}⩽$$

$$⩽\lim_{n\to \infty }\sum_{W=1}^{\left\lfloor \varepsilon^{\prime }n\right\rfloor }{\left({\left(\varepsilon^{\prime }\right)}^{c_{1}}2^{-c_{2}}\right)}^{W}=\frac{{\left(\varepsilon^{\prime }\right)}^{c_{1}}2^{-c_{2}}}{1-{\left(\varepsilon^{\prime }\right)}^{c_{1}}2^{-c_{2}}}=\varepsilon^{\prime \prime }.$$

It should be noted that the sign of $c_{2}$ is not important because $\varepsilon^{\prime \prime }$ can be made arbitrarily small by a correct choice of $\varepsilon^{\prime }$.

Thus,

$$\lim_{n\to \infty }\sum_{W=1}^{\left\lfloor \varepsilon^{\prime }n\right\rfloor }2^{-n\left(\ell F_{e}\left(\frac{W}{n},n_{0}\right)-h\left(\frac{W}{n}\right)\right)}⩽\varepsilon^{\prime \prime }<1.$$

Consequently, the code for which the condition (11) is satisfied for all values of $\omega_{t}<\varepsilon^{\prime }$ exists with non-zero probability in the ensemble of G-LDPC codes.  □

Finally, let us consider the proof of Corollary 1.

**Proof.**  The correctness of the corollary is easy to see if we note that $E_{0}\left(\cdot \right)>0$ for $R_{1}<C$ [6] and $E_{2}\left(\cdot \right)\geq 0$, which follows from (6), and we can always select $n_{1}$ such that $\frac{1}{n_{1}}H\left(\beta \right)<\beta E_{0}\left(\cdot \right)+E_{2}\left(\cdot \right)0pt12.5pt$ because $n_{1}$ can be arbitrarily large according to condition (3). Therefore, according to Corollary 2, the construction of G-LDPC code with $\omega_{t}>0$ for any code rate $R_{2}<1$ exists, helping us omit this condition in the corollary formulation (unlike the formulation of a similar corollary in [12]).  □

### 7.2. Error Exponent for Decoding Algorithm $A_{ML}$

Let us consider the proof of Theorem 2.

**Proof.**  To simplify the proof without loss of generality, let us consider the transmission of a zero codeword over the BSC with BER *p*. Let the probability of the transition of the zero codeword to each of $N\left(w\right)$ codewords with weight *w* during decoding with algorithm $A_{ML}$ be equal to $P_{\delta }\left(w\right)$. Moreover, let there exist a critical value $w_{c}$ of the number of errors that leads to erroneous decoding with algorithm $A_{ML}$. Then, we can write

$$P_{ML}=\sum_{w=d}^{w_{c}}N\left(w\right)P_{\delta }\left(w\right)+P\left(w\geq w_{c}\right),$$

where *d* is the code distance of the LG-LDPC code.

To obtain the upper bound, it is sufficient to consider the case when the zero codeword becomes the word with weight *w* if there are more than w/2 errors:

$$P_{\delta }\left(w\right)=\sum_{i=\frac{w}{2}}^{w}\left(\begin{array}{c} w\\ i \end{array}\right)p^{i}{\left(1-p\right)}^{w-i}\leq 2^{w-1}p^{\frac{w}{2}}{\left(1-p\right)}^{\frac{w}{2}},$$

where $p\leq \frac{1}{2}$.

From this inequality, we easily obtain

$$E_{\delta }\left(\omega_{c},p\right)=\max_{\delta \leq \omega \leq \omega_{c}}\left\{\nu \left(\omega \right)+\omega \left(\ln2+\ln\sqrt{p\left(1-p\right)}\right)\right\},$$

where $\nu \left(\omega \right)$ is an asymptotic spectrum of the LG-LDPC code given by Lemma 1 and δ is the relative code distance of the LG-LDPC code given by Lemma 2.

With the help of the Chernoff bound, we obtain the exponent of the probability that more than $w_{c}$ errors have occurred:

$$P\left(w\geq w_{c}\right)\leq \exp\left\{-nE_{\omega_{c}}\left(\omega_{c},p\right)\right\}.$$

$$E_{\omega_{c}}\left(\omega_{c},p\right)=\left(1-\omega_{c}\right)\ln\frac{1-\omega_{c}}{1-p}+\omega_{c}\ln\frac{\omega_{c}}{p},\omega_{c}\geq p.$$

Consequently,

$$E_{ML}\left(p\right)=\max_{\omega_{0}\leq \omega_{c}\leq 1}\left\{\min\left(E_{\delta }\left(\omega_{c},p\right),E_{\omega_{c}}\left(\omega_{c},p\right)\right)\right\},$$

$$\omega_{0}=\max\left(\delta ,p\right).$$

 □

The estimations given in Lemmas 1 and 2 were obtained by the slightly modified classical Gallager’s method [1]. Thus, in this paper, we give only a sketch of the proof.

Let us first consider the proof of Lemma 1.

**Proof.**  Let us consider the fixed word of weight *W* and find the probability of there being a code in the LG-LDPC code ensemble such that this word is a codeword for this code. For this purpose, let us consider the first layer of the parity-check matrix of some LG-LDPC code from the ensemble composed of the parity-check matrices of the single parity check code. We can write the probability that the considered word is a codeword for a given layer as follows:

PW1=N1WnW,

where $N_{1}\left(W\right)$ is the number of layers, and the word of weight *W* is a codeword.

We estimate $N_{1}\left(W\right)$ as

$$N_{1}\left(W\right)⩽\min_{s>0}\left\{\frac{g_{0}^{b_{0}}\left(s,n_{0}\right)}{s^{W}}\right\},$$

where $g_{0}\left(s,n_{0}\right)$ is a spectrum function of the SPC code.

Thus,

PW1⩽nw−1mins>0g0b0s,n0s−W.

It is clear that the obtained estimation is the same for all ℓ−1 layers:

PWi⩽nw−1mins>0g0b0s,n0s−W,i=1…ℓ−1.

Similarly, we can write the probability that the considered word of weight *W* is a codeword for the *ℓ*th layer of the parity-check matrix composed of “optimal” linear codes:

PWℓ⩽nw−1mins>0g1b1s,R1,n1s−W,

where $g_{1}\left(s,R_{1},n_{1}\right)$ is a spectrum of the code with a good spectrum.

Since the layer permutations are independent, we can write the probability that the given word of weight *W* is a codeword for the whole code construction as

PW=∏i=1ℓPWi⩽nW−ℓmins>0g0b0ℓ−1s,n0g1b1s,R1,n1s−Wℓ.

Consequently, the average number of weight *W* codewords is given by

N¯W=nWPW⩽nW−ℓ−1mins>0g0b0ℓ−1s,n0g1b1s,R1,n1s−Wℓ.

For W=ωn, we obtain

$$\nu \left(\omega \right)=\lim_{n\to \infty }\frac{\ln N\left(\omega n\right)}{n}⩽\nu_{0}\left(\omega \right).$$

 □

Now, let us consider the proof of Lemma 2.

**Proof.**  If the average number of codewords $\bar{N}\left(W\right)$ in the ensemble of LG-LDPC codes satisfies the condition

$$\sum_{W=1}^{d_{0}}\bar{N}\left(W\right)⩽1,$$

then the code with code distance $d⩾d_{0}$ exists in this ensemble.

It is easy to show that the sum of the right part of the inequality can be estimated with the last member of this sum. Therefore, using the estimation obtained in the previous lemma, we can write

$$\nu_{0}\left(\delta \right)⩽0,$$

where δ=d/n is the relative code distance.

Thus, we can obtain the maximum value of $\delta_{0}$ such that the above-considered condition is satisfied for all smaller values $\delta ⩽\delta_{0}$0 as the smallest positive root of the following equation:

$$\nu_{0}\left(\delta_{0}\right)=0.$$

 □

## Data Availability

Not applicable.
